# Lithium use associated with symptom severity in comorbid bipolar disorder I and migraine

**DOI:** 10.1002/brb3.2585

**Published:** 2022-05-05

**Authors:** Nicole M. Sekula, Anastasia K. Yocum, Steven Anderau, Melvin G. McInnis, David F. Marshall

**Affiliations:** ^1^ University of Michigan Medical School Ann Arbor Michigan USA; ^2^ Department of Psychiatry University of Michigan Ann Arbor Michigan USA

**Keywords:** bipolar disorder, lithium, mania, migraine

## Abstract

**Introduction:**

Bipolar disorder (BD) and migraine headaches are frequently comorbid. The common etiological features are unknown, however cortical hyperexcitability (EEG) of migraines, and the report of hyperexcitability in pluripotent stem cell‐derived neurons from lithium responsive BD subjects offers a physiological hypothesis of excitable neurons linking these disorders. However, clinical studies suggest that a history of migraine is associated with higher rates of relapse in those with BD taking lithium. Lithium use and history of migraine in this prospective longitudinal study of BD find that lithium use is associated with a greater symptom severity in BD.

**Methods:**

Data on longitudinal outcome from 538 patients with BD I were categorized according to treatment with lithium and comorbidity with migraine. Clinical outcome measures on depression, mania, and quality of life over the most recent 2‐year period compared the BD and BD/migraine cohort according to lithium treatment status.

**Results:**

A history of migraines was associated with worse clinical outcomes of depression (*p* = .002), mania (*p* = .005), and mental and physical quality of life (*p* = .004 and *p* = .005, respectively), independent of lithium use. The BD/migraine cohort treated with lithium was associated with worse symptoms of mania, whereas those without migraine and lithium use were associated with milder manic symptoms (*p* = .026).

**Conclusions:**

Herein, we replicate the relatively worse outcome in BD with comorbid migraine. We find evidence to suggest that lithium use is associated with more severe symptoms of mania among those with BD and a history of migraine and conclude that lithium is contraindicated in BD comorbid with migraine.

## Graphical abstract

A history of migraines was associated with worse clinical outcomes of depression, mania, and mental and physical quality of life, independent of lithium use. The bipolar disorder/migraine cohort treated with lithium was associated with worse symptoms of mania, whereas those without migraine and lithium use was associated with milder manic symptoms. We find evidence to suggest that lithium use is associated with more severe symptoms of mania among those with bipolar disorder and a history of migraine.

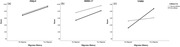

## 1 INTRODUCTION

Bipolar disorder (BD) is characterized by pathological mood swings ranging from mania to depression. Individuals with BD often have comorbid medical disorders, with migraines being one of the most common (Oedegaard et al., 2011; Sinha et al., 2018). The prevalence of migraines in the general population ranges between 12% and 22% (Smitherman et al., 2013), and the prevalence of migraines in individuals with BD is approximately 30% (Leo & Singh, 2016). Comorbid migraine in BD is associated with worse clinical outcomes, particularly with regards to depression severity (Mehrhof et al., 2020; Saunders et al., 2014), as well as suicidal behavior and comorbid anxiety disorders (Ortiz et al., 2010).

The connection between BD and migraines may be in part mediated by neuronal excitation. Previous genome‐wide association studies have found changes in several genes that may contribute to the correlation between BD and migraines, including the Na^+^/Ca^2+^ exchanger gene (Oedegaard et al., 2010), the NBEA gene involved in glutamate signaling (Jacobsen et al., 2015), and the gene encoding the alpha isomer of the Na^+^/K^+^ ATPase protein (Retamales‐Ortega et al., 2016). These genes all encode for proteins involved in excitatory signaling in the brain, suggesting that neuronal network excitability may play a role in the comorbidity of BD and migraine. Of note, individuals with migraine have a lower threshold for excitation by transcranial magnetic stimulation (TMS) compared to controls, making their neurons more sensitive to excitation (Aurora et al., 1998), consistent with the neuronal hyperexcitation hypothesis of migraines (Scheffer et al., 2013).

Cellular models also suggest that changes in neuronal excitability are associated with lithium response in BD as well (Scott et al., 2020; Stern et al., 2018). In iPSC models of BD, neurons derived from lithium responsive individuals have a higher spontaneous firing rate (i.e., excitability) and higher sodium current than lithium nonresponder groups (Stern et al., 2018). In these studies, lithium treatment decreased Na^+^/K^+^ currents and total action potentials in lithium responder neurons but not lithium nonresponder neurons. Furthermore, neurons from individuals with BD appear to be hyperexcitable only if derived from lithium responders (Stern et al., 2020). Additionally, genetic variants of the alpha isoform of Na^+^/K^+^ ATPase have been found in those with BD as well as those with familial hemiplegic migraine, and lithium subsequently decreases Na^+^/K^+^ ATPase inhibition (Retamales‐Ortega et al., 2016). This suggests that neuronal hyperexcitability in BD may be altered by lithium and the degree of excitability may ultimately predict lithium response.

Despite the physiological parallels in neuronal excitability patterns between individuals with migraine and lithium responders in BD, a recent study reported that a history of migraine is associated with higher rate of relapse during lithium treatment (Lin et al., 2021). This relationship between lithium response and migraine history seems contradictory given the putative underlying neuronal mechanisms connecting the two conditions. Nonetheless, this current clinical literature suggests that there may be a limited use of lithium when migraine coexists with BD. Given the changes in neuronal excitation in the pathology of BD and migraine, the altered neuronal excitation pathways in lithium responder groups, and the seemingly paradoxical effect of migraine history on lithium response in BD observed clinically, we sought to further explore the relationship between lithium responsiveness in patients with BD and comorbid migraines. Our study looked at measures of depression, mania, and mental/physical health in those with BD with a history/no history of migraine and current lithium use. This was tested in an established longitudinal cohort.

## 2 MATERIALS AND METHODS

### 2.1 Study participants

Participants were recruited for in the HC Prechter Bipolar Research Program between October 2005 and December 2016 at the University of Michigan for an outcomes study of BD (for a description, see Mcinnis et al., 2018). This study was approved by the University of Michigan Institution Review Board. Only participants that met criteria for a BD I diagnosis and had longitudinal data were included in the present study. We analyzed the longitudinal data of 538 individuals with confirmed BD I. Of these, 169 (31%) had a history of migraines, and 369 (69%) did not report a history of migraine. We utilized data that included each participants’ most recent follow‐up evaluation and the 2 years prior to that evaluation, with four comparison groups created: BD with migraine on lithium (*n* = 26), BD with migraine not taking lithium (*n* = 143), BD without migraine on lithium (*n* = 71), and BD without migraine not taking lithium (*n* = 298). Lithium treatment status was defined by medication use reported at participants’ most recent follow‐up evaluation. Participants were evaluated as described in Mcinnis et al. (2018). The data for this current study are from the Diagnostic Interview for Genetic Studies (DIGS) (Nurnberger, 1994), Hamilton Depression Rating Scale‐17 (HDRS‐17) (Hamilton, 1960), and Young Mania Rating Scale (YMRS) (Young et al., 1978). Participants completed serial Patient Health Questionnaires‐9 (PHQ‐9) (Kroenke et al., 2001), and the 12‐Item Short Form Surveys (SF‐12) (Ware, 2000) at 2‐month intervals and the HDRS and YMRS yearly. Migraine history was captured with the medical history section (Section B) of the DIGS which was completed during their initial enrollment.

### 2.2 Data analysis

We used IBM SPSS 26 for all statistical analyses. Analysis of variance (ANOVAs) and/or independent samples *t*‐tests were used to assess between‐group differences in continuous demographic variables. Chi‐square analyses were utilized to assess differences in categorical demographic variables. Analysis of covariance (ANCOVAs) was used to compare mean score of each clinical assessment over the previous 2 years (prior to their most recent follow‐up evaluation) between four groups (migraine taking lithium, migraine not taking lithium, no migraine taking lithium, and no migraine not taking lithium), with gender as a covariate and with subsequent planned pairwise comparison. Scores of each clinical assessment were averaged over the past 2 years prior to their most recent evaluation. The alpha level was set at *p* < .05.

## 3 RESULTS

### 3.1 Sample characteristics

Table 1 contains the clinical characteristics of participants in the migraine and no migraine groups. There was no difference in age (BD migraine mean = 46.8, BD no migraine = 48.5), race (BD migraine % Caucasian = 82.8%, BD no migraine % Caucasian = 88.6), and length of time in study (BD migraine = 6.42 years, BD no migraine = 6.84 years). There was a significant difference in gender across groups: The migraine group had significantly more females than the no migraine group (*p* < .001), consistent with previous findings of sex in migraine prevalence (Finocchi & Strada, 2014). The migraine group was more likely to take benzodiazepines (*p* = .023) and trended toward more antipsychotic medication use (*p* = .075). Use of other medications did not differ significantly.

**TABLE 1:** Demographic and clinical characteristics in individuals with bipolar disorder (BD) with and without a history of migraine

	Migraine (*n* = 169) *M* (SD)	No migraine (*n* = 369) *M* (SD)	*p*‐Value
Age	46.8 (13.0)	48.5 (13.9)	.193
Gender (M/F)	33/132	167/199	<.001^***^
Race (% White)	82.8%	88.6%	.297
Length in study (years)	6.42 (3.6)	6.84 (4.0)	.243
PHQ‐9	9.33 (5.5)	7.00 (5.4)	<.001^***^
YMRS	3.25 (4.5)	2.37 (4.0)	.032^*^
SF‐12 MCS	37.67 (10.0)	40.97 (11.1)	.003^**^
SF‐12 PCS	42.83 (11.6)	47.14 (10.0)	<.001^***^
HDRS‐17	9.00 (7.7)	7.04 (6.7)	.007^**^
Medication^1^			
Lithium	26 (15.4%)	71 (19.2%)	.280
Amphetamines	12 (7.1%)	21 (5.7%)	.527
Anticonvulsants	68 (40.2%)	143 (38.8%)	.744
Antidepressants	66 (39.1%)	124 (33.6%)	.220
Antipsychotic	46 (27.2%)	129 (35.0%)	.075
Benzodiazepines	43 (25.4%)	63 (17.1%)	.023^*^
Migraine medication	13 (7.7%)	4 (1.1%)	<.001^***^

Abbreviations: BD, bipolar disorder; HDRS‐17, Hamilton Depression Rating Scale 17‐item; PHQ‐9, Patient Health Questionnaire‐9; SF‐12 MCS, 12‐item short form survey mental component; SF‐12 PCS, 12‐item short form survey physical component; YMRS, Young Mania Rating Scale.

^*^ *p* < .05.

^**^ *p* < .01.

^***^ *p* < .001.

^1^ *N* (% of migraine/no migraine‐specific cohort).

### 3.2 Assessment and measures

#### 3.2.1 Relationship between groups and clinical outcome measures

ANCOVAs were computed with the clinical outcome measures as the dependent variable and the four groups as independent variables. Table 2 contains the mean values and standard deviations between the four groups for each of the clinical outcome measures. There was a significant group difference for all mood and quality of life measures, including PHQ‐9, *F* (3, 459) = 5.80, *p *= .001, YMRS, *F* (3, 489) = 3.04, *p *= .029, SF‐12 MCS, *F* (3, 432) = 2.95, *p *= .032, SF‐12 PCS, *F* (3, 432) = 8.31, *p *< .001, and HDRS‐17, *F* (3, 401) = 3.61, *p *= .013.

**TABLE 2:** Association between migraine, lithium, and the migraine–lithium interaction on clinical scores

	PHQ mean (SD)	YMRS mean (SD)	SF‐12 MCS mean (SD)	SF‐12 PCS mean (SD)	HDRS‐17 mean (SD)
No migraine + no lithium	7.11 (5.3)	2.61 (4.2)	40.63 (11.1)	46.42 (10.5)	7.52 (6.7)
No migraine + lithium	6.67 (5.5)	1.50 (2.6)	42.18 (11.5)	49.90 (7.3)	5.33 (6.4)
Migraine + no lithium	9.45 (5.6)	3.04 (4.3)	38.14 (9.8)	41.88 (11.5)	9.32 (8.1)
Migraine + lithium	9.34 (5.5)	4.42 (5.5)	35.53 (10.0)	46.17 (12.0)	8.16 (5.9)
*p*‐Value (ANCOVA)					
Overall	.001^**^	.029^*^	.032^*^	<.001^***^	.013^*^
Migraine	.002^**^	.005^**^	.004^**^	.005^**^	.032^*^
Lithium	.777	.752	.658	.007^**^	.111
Migraine × lithium	.871	.026^*^	.180	.788	.624

Abbreviations: ANCOVA, analysis of covariance; HDRS‐17, Hamilton Depression Rating Scale 17‐item; PHQ, Patient Health Questionnaire; SF‐12 PCS, 12‐item short form survey physical component; YMRS, Young Mania Rating Scale.

^*^ *p* < .05.

^**^ *p* < .01.

^***^ *p* < .001.

#### 3.2.2 Relationship between migraine and lithium on clinical outcome measures

**FIGURE 1:**
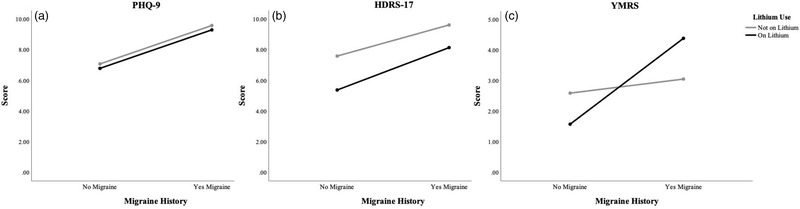
Effect of migraine, lithium and the migraine‐lithium interaction on Patient Health Questionnaires‐9 (PHQ) (a), Young Mania Rating Scale (YMRS) (b), and Hamilton Depression Rating Scale 17‐item (HDRS‐17) (c)

**FIGURE 2:**
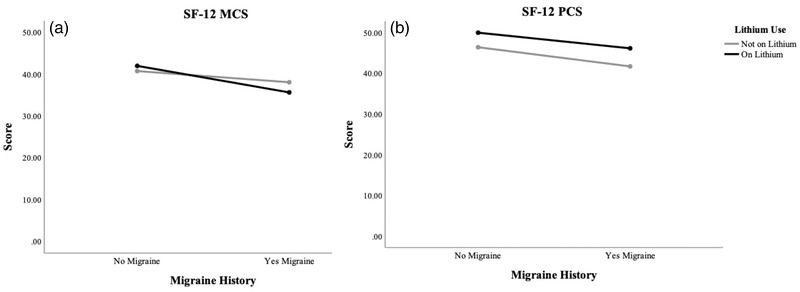
Effect of migraine, lithium, and the migraine–lithium interaction on mental (a) and physical (b) quality of life

There was a significant effect of history of migraine on all clinical outcome measures, including the PHQ‐9, *F* (1, 459) = 10.016, *p *= .002, YMRS, *F* (1, 489) = 8.13, *p *= .005, SF‐12 MCS, *F* (1, 432) = 8.26, *p *= .004, SF‐12 PCS, *F* (1, 432) = 8.08, *p *= .005, and HDRS‐17, *F* (1, 401) = 4.65, *p *= .032 (Table 2, Figures 1a–c and 2a,b). Specifically, those with a history of migraine had more severe mood scores, indicative of a more serious disease outcome, and lower quality of life scores compared to those with no history of migraine. There was also a significant effect of lithium on the SF‐12 PCS, *F* (1, 432) = 7.24, *p *= .007, where those taking lithium had higher physical health quality of life scores compared to those not on lithium, independent of migraine (Table 2, Figure 2a,b).

A significant interaction effect was found between migraine and lithium on the YMRS, *F* (1, 489) = 4.98, *p *= .026, which is illustrated in Figure 1. Those with and without a history of migraine were affected differently by lithium use/nonuse. Specifically, those with migraine taking lithium had higher YMRS scores (*M* = 4.42, SD = 5.47) compared to those with migraine not taking lithium (*M* = 3.04, SD = 4.33), those without migraine taking lithium (*M* = 1.5, SD = 2.6), and those without migraine not taking lithium (*M* = 2.61, SD = 4.23). This interaction remained even after adding the additional covariate of depression, *F* (1, 434) = 5.55, *p *= .019.

## 4 DISCUSSION

Our investigation into the clinical connection between lithium treatment in patients with BD I and comorbid migraines yielded two key findings. First, those with BD I and a history of migraines report worse clinical outcomes overall compared to those without migraines—for symptoms of depression, mania, and both physical and mental quality of life. This is consistent with previous studies finding increased mixed features, risk for suicidality (Fornaro et al., 2015), increased mood episodes, and a higher prevalence of psychiatric and general medical comorbidities (Brietzke et al., 2012) in those with comorbid migraine and BD (Ortiz et al., 2010). Second, those who have a history of migraine and are taking lithium report more symptoms of mania than those with no history of migraine taking lithium. Given that the YMRS score was low in all groups, participants with a history of migraine who were taking lithium were likely not experiencing florid mania but rather subtle symptoms of elevated mood. Additionally, while studies investigating clinical outcomes in those with BD and migraine focus primarily on depressive symptoms (Mehrhof et al., 2020; Saunders et al., 2014), our current work fills this gap by investigating this effect for symptoms of mania. Our results also showed a correlation between lithium use and increased physical quality of life and decreased depressive symptoms independent of migraines, which was expected.

Lithium is considered a first‐line treatment for BD, and efforts are ongoing to identify predictors of lithium and other medications to improve medication guidelines (Yatham et al., 2018). A recent published study done by Lin et al. (2021) found that those with BD and a history of migraine taking lithium had higher rates of relapses—defined by episodes of mania, depression, mixed episodes, hospitalization, or the need for medication changes in order to obtain better mood stabilization—than those with BD and no history of migraine taking lithium. Our research expands these findings by identifying the specific mood state (mania) that is affected by this interaction. Given the similarly hyperexcitable molecular profiles of neurons in those with migraine and those who are lithium responsive, these findings suggest a paradoxical effect of lithium on those with BD and migraines. Ultimately, migraine as a comorbid feature of BD may identify individuals at greater risk for symptoms of mania on lithium.

Although there is increased hyperexcitability of neurons in those who are lithium responders (Stern et al., 2018) as well as those with a history of migraine (Aurora et al., 1998), these different excitability patterns are seen in different parts of the brain. For lithium responders, hippocampal neurons were utilized and for those with a history of migraine, cortical neurons were tested with TMS. It may therefore be that neuronal excitability of cortical neurons in migraine does not convey increased hippocampal neuronal excitability seen in iPSC models of lithium responsive BD. While our study did not directly test the neurobiological links involved in the lithium–migraine interaction, future studies might consider the different patterns of neuronal hyper/hypoactivity between these two groups, particularly during episodes of mania.

This study has several limitations. Self‐reported data collection for migraine history and lithium treatment was based on clinical interviews and not verified in medical records. However, while the validity of self‐reported medical data has been questioned, it has been shown to be acceptable for chronic conditions (Martin et al., 2000). Additionally, we did not determine if participants were lithium responders per se, they were simply designated as taking lithium or not and their symptom severity scores were interpreted in the context of lithium/no lithium use. Blood lithium levels would have been beneficial to obtain from participants but given the method of data collection in our study, we did not have access to this information. Furthermore, the timing of migraine onset and onset of lithium use is unknown. Future studies could expand upon this investigation by obtaining serum lithium levels and clarifying the timing of migraine/lithium use onset. The migraine group was also more likely to take benzodiazepines, but the use of short‐acting benzodiazepines has been shown to increase migraine occurrence (Harnod et al., 2017).

Our findings provide a compelling argument for focused research on comorbid migraine and BD as the co‐occurrence of these two disorders is substantial. There is convincing evidence that those with BD and migraine have greater burden of disease that may in fact not benefit from treatment with lithium. If these findings are supported further, it would be worthy to consider that lithium is, in fact, contraindicated in BD comorbid with migraine.

In conclusion, this study found significant differences in the clinical characteristics of those with no history of migraine compared to those with a history of migraine in BD I, particularly with regard to manic symptomology and self‐reported physical and mental quality of life. Additionally, our findings worse symptoms of mania in those with comorbid BD/migraine who were on lithium treatment highlight the importance of a thorough neurological history and review of headache patterns that are consistent with migraine. Our findings along with published observations suggest that lithium is in fact contraindicated in comorbid BD/migraine and other mood stabilizing medications should be considered.

## CONFLICT OF INTEREST

Melvin G. McInnis has consulted with Janssen and Otsuka pharmaceuticals, and received research support from Janssen.

### PEER REVIEW

The peer review history for this article is available at https://publons.com/publon/10.1002/brb3.2585.

## Data Availability

All data and samples are available through the Heinz C. Prechter Genetic Repository, distributed by the University of Michigan Central Biorepository (CBR). Enquiries can be addressed at http://www.prechterprogram.org/data. Longitudinal and outcomes data are available subject to review of the proposed analyses and acceptance of a Data Use Agreement.
